# Identifying of Anti-Thrombin Active Components From Curcumae Rhizoma by Affinity-Ultrafiltration Coupled With UPLC-Q-Exactive Orbitrap/MS

**DOI:** 10.3389/fphar.2021.769021

**Published:** 2021-12-10

**Authors:** Zhenwei Lan, Ying Zhang, Yue Sun, Lvhong Wang, Yuting Huang, Hui Cao, Shumei Wang, Jiang Meng

**Affiliations:** ^1^ School of Traditional Chinese Medicine, Guangdong Pharmaceutical University, Key Laboratory of Digital Quality Evaluation of Chinese Materia Medica, State Administration of Traditional Chinese Medicine (TCM), Engineering Technology Research Center for Chinese Materia Medica Quality of Universities in Guangdong Province, Guangzhou, China; ^2^ College of Pharmacy, Jinan University, Research Center for Traditional Chinese Medicine of Lingnan, Guangdong Provincial Key Laboratory of Traditional Chinese Medicine Informatization, Guangzhou, China

**Keywords:** curcumae rhizoma, affinity-ultrafiltration-MS, diarylheptanoid, antithrombosis, zebrafish, thrombin inhibitors

## Abstract

Recent studies concerning products that originate from natural plants have sought to clarify active ingredients, which both explains the mechanisms of the function and aids in quality control during production. As a traditional functional plant, Curcumae Rhizoma (CR) has been proven to be effective in promoting blood circulation and removing blood stasis. However, the components that play a role in its huge compound library are still unclear. The present study aimed to develop a high-throughput screening method to identify thrombin inhibitors in CR and validate them by *in vitro* and *in vivo* experiments. The effect of CR on thrombin in HUVECs cells was determined by ELISA, then an affinity-ultrafiltration-UPLC-Q-Exactive Orbitrap/MS approach was applied. Agatroban and adenosine were used as positive and negative drugs respectively to verify the reliability of the established method. The *in vitro* activity of the compounds was determined by specific substrate S-2238. The *in vivo* effect of the active ingredients was determined using zebrafish. Molecular docking was used to understand the internal interactions between compounds and enzymes. ELISA results showed that CR had an inhibitory effect on thrombin. The screening method established in this paper is reliable, by which a total of 15 active compounds were successfully identified. This study is the first to report that C7, 8, and 11 have *in vitro* thrombin-inhibitory activity and significantly inhibit thrombosis in zebrafish models at a safe dose. Molecular docking studies were employed to analyze the possible active binding sites, with the results suggesting that compound 16 is likely a better thrombin inhibitor compared with the other compounds. Based on the affinity-ultrafiltration-UPLC-Q-Exactive Orbitrap/MS approach, a precisely targeted therapy method using bio-active compounds from CR might be successfully established, which also provides a valuable reference for targeted therapy, mechanism exploration, and the quality control of traditional herbal medicine.

## Introduction

Thrombus, as one of the most commonly observed etiological factors causing a variety of cardiovascular and cerebrovascular diseases such as hypertension and cerebral ischemic stroke, has been attributed to the injury of vascular endothelial cells, including serious injuries related to surgery and childbirth, as well as changes in blood rheology and other pathological changes (Naghavi et al., 2017; Zhao et al., 2019). By abnormally activating the coagulation pathways, these pathological changes disturb the normal clotting mechanisms, ultimately leading to unnecessary thrombus formation. Unfortunately, as we age, the potential risk of thrombogenesis is ever increasing due to higher exposure to these changes, along with the aging of blood vessels. Many scholars have dedicated themselves to the exploration of suitable targets in coagulation pathways and attempted to develop a solution that aids the treatment and prevention of thrombosis-relevant diseases by manipulating these targets (Liu et al., 2021).

Thrombin (FIIα), a key enzyme in thrombosis, is a downstream factor of the coagulation pathway. *In vivo*, it converts fibrinogen into fibrin monomer, or factor XIII into factor XIIIα, thus binding with calcium ions to form a fibrin network, which is already known to be a critical link in thrombosis. Therefore, great attention has been paid to thrombin as an antithrombotic target. Vorapaxar was the first thrombin receptor inhibitor (THRI) approved by the Food and Drug Administration (FDA) in 2014 (Poole and Elkinson, 2014), but clinical trials have shown that its use increases the rate of severe bleeding, including intracranial haemorrhage in patients with a history of stroke (Vranckx et al., 2016). The research and development of Atopaxar (O’Donoghue et al., 2011) PZ-128 became trapped in a dilemma during phase II clinical trials for similar reasons (Gurbel et al., 2016). Part of the latest generation of oral direct thrombin inhibitors, Dabigatran (Pradaxa) has minimal side effects with other foods and drugs, a rapid clotting effect, and a wide treatment window (Lee and Ansell, 2011; van Ryn et al., 2013); however, there is still a risk of causing life-threatening bleeding after kidney damage (Summers and Sterling, 2016). These setbacks in antithrombosis studies have led researchers to seek safer sources, for instance, the vast compound library of natural products, aiming to anchor some new alternatives to those thrombin inhibitors mentioned above, since potential active components with a thrombin-inhibitory effect, such as salvianolic acid A, B, C, and protocatechuic acid, have been obtained from natural products in previous studies (Cao et al., 2016; Wu et al., 2020).

Natural plants have been used, especially in China, Japan, and Southeast Asia, as a functional food and phytomedicine for thousands of years (Liu and Nair, 2012; Zhang L. et al., 2017). Although many of the specific pharmaco-mechanisms remain unexplored, their long-term use has been well documented, which lays a solid foundation for further exploration (Zhou et al., 2016). According to the Chinese Pharmacopoeia, Curcumae Rhizoma (CR), the dried rhizome of *Curcuma phaeocaulis* Val., *Curcuma kwangsiensis* (S.G. Lee and C.F. Liang) or *Curcuma wenyujin* (Y.H. Chen and C.Ling), which is a synonym for *Curcuma aromatica* Salisb., has long been used in China and Japan as a medicinal plant for promoting blood circulation. It has two existing medicinal products—raw CR (RCR) and vinegar-processed CR (PCR), both bearing, but in various intensity, an effect of promoting blood circulation and removing blood stasis (Chinese Pharmacopoeia Commission, 2020). Previous network pharmacological studies have preliminarily revealed that CR may have an active effect on thrombin receptor (THR) (Tao et al., 2013). A recent study in our laboratory with a representative sample size showed that RCR was generally stronger *in vitro* than PCR in inhibiting THR, and this inhibition was highly correlated with the near infrared ray (NIR) spectrum, which reflects the overall chemical composition of CR products. This study, for the first time, proved the inhibitory effect of CR products on THR (Lan et al., 2021). However, the specific molecular mechanism of their THRI activity is still not clear, thus necessitating deeper research to further identify and verify the molecular compositions of the active substances in CR products.

In recent studies, affinity ultrafiltration coupled with ultra-performance liquid chromatography-mass spectrometry (AUF-LC-MS) has proved to be effective for the rapid characterization of the target molecules in a given compound (Xie et al., 2020, 2021). Although the spectrum-effect relationship analysis is also a useful screening method, false positive results may easily occur if the toxicity of the compound to the enzyme is considered. In the process of AUF-LC-MS identification, AUF can screen ligand-protein complexes from unbound substances, while LC-MS can identify target substances with varying contents. Being a high throughput method, AUF-LC-MS has a good performance in active substance screening without a high demand in sample size, in addition to some other benefits such as simple operation and strong targeting. Besides, the formation of protein-ligand complexes takes place in a condition that mimics the degrees of freedom in the actual biological system, making this screening method more practical and reliable (Wei et al., 2016). However, there are also some problems associated with this technique, which often compromise the experimental results. For instance, positive drugs are typically used to explore the AUF conditions (Wang S. et al., 2020), whereas, the drugs, as a compound monomer, usually cannot well represent a condition that simulates the rich compounds library in natural plants. In addition, false-positive results caused by various factors are usually formed during the dissociation of ligand-protein complexes. As a solution, the chromatic substrate method was applied in this study to explore the experimental conditions of AUF for the total extract. Meanwhile, unbound fraction analysis (UFA), an approach validated to be effective in a variety of previous studies (Tao et al., 2015), was also employed here by comparing it with bound fraction analysis (BFA) (Qin et al., 2015), with the deactivated-THR experimental group established as the control to analyze the filtrate and reduce the impact of non-specific binding.

In summary, to further explore the internal mechanism of CR in promoting blood circulation and removing blood stasis, an appropriate AUF-LC-MS method was developed in this study to identify potential inhibitors from CR extracts. As a result, a series of diarylheptanoid compounds were identified to be active in thrombin inhibition. To the best of our knowledge, the activity of diarylheptanoid compounds against THR has not been previously reported, therefore, this finding reveals potential new applications for these compounds. In the present study, the *in vivo* inhibition ability of the samples was also evaluated using established zebrafish thrombosis models. Molecular docking technology was used to preliminarily explore the binding mechanism between the active molecules and THR.

## Materials and Methods

### Materials and Animals

The reagents used in this study and the sources were as follows: human recombinant THR, Yeasen Biotech Co., Ltd. (Shanghai, China); chromogenic substrate S-2238 (98%), Yuanye Bio-technology Co., Ltd. (Shanghai, China); phosphate buffer saline (PBS, pH = 6.5), CORNING, Inc. (New York, United States); 4,4′-[3,5-bis(acetyloxy)-1,7-​heptanediyl]bis-1,2-benzenediol (C7), 3-acetate-1,7-bis(4-hydroxyphenyl)-3,5-heptanediol (C8), and 4-[3,5-bis(acetyloxy)-7-(4-hydroxyphenyl)heptyl]-1,2-benzenediol (C11), Grint Biological Technology Co., Ltd. (Wuhan, China); arachidonic acid (AA, 99%, No. C2123090), aspirin (99%, No. H2017088) and O-dianisidine (3,3′Dimethoxybenzidine, 97%, No. C2009167), Shanghai Aladdin Biochemical Technology Co., Ltd. (Shanghai, China). All of the reagents were of corresponding analytical grade.

Centrifugal ultrafiltration filters (Amicon Ultra-0.5, 10 kDa) was purchased from Millipore Co., Ltd. (Bedford, MA, United States). FIIα ELISA kit was supplied by Shanghai Fusheng Biotechnology Co., Ltd., (Shanghai, China).

The RCR (batch number: 200101231) and PCR samples (batch number: 191200361) were procured from Kangmei Pharmaceutical Co., Ltd., (Guangdong, China) and identified by Professor Jizhu Liu from the School of Traditional Chinese Medical Materials, Guangdong Pharmaceutical University. Voucher specimens were deposited at the Herbarium Centre, Guangdong Pharmaceutical University. To facilitate extraction, the samples were crushed in a rocking pulverizer (DFY-400-D) and passed through an 80 mesh sieve, and then dried at 45°C and sealed for preservation. Liquid extract was prepared from CR powder using methanol (1:3, w/v) with sonication, and finally, a rotary evaporator and freeze dryer were used to obtain the CR extract, which was then sealed and stored at 4°C.

Zebrafish AB strains from the National Zebrafish Resource Center were used in the antithrombotic activity experiment. Tail thrombus staining intensity reportedly, which has a high negative correlation with cardiac staining intensity (Zhu et al., 2016), was used to evaluate the degree of thrombosis (Jagadeeswaran et al., 2016; Wang AK. et al., 2020). The zebrafish were fed on live brine shrimp twice a day in an automatic circulating tank system in the key laboratory of digital quality evaluation of Chinese Materia Medica, Guangdong Pharmaceutical University (Guangzhou, China), with the condition controlled steadily for a 14 h light/10 h dark cycle. Water temperature was maintained at 28 ± 0.5°C and pH at 7.0 ± 0.5. The embryos were produced naturally by the zebrafish. After spawning, the fertilized eggs were collected and washed with culture water 3 times. Then, they were placed in a 28°C-light incubator, and the activity evaluation experiment was conducted with 3 dpf fish. All the animal procedures in our study were carried out according to the Regulations of Experimental Animal Administration issued by the State Committee of Science and Technology of China and approved by the institutional ethical committee (IEC) of Guangdong Pharmaceutical University.

### Determination of thrombin Inhibitory Activity

The thrombin inhibition assay was performed based on a previous examination (Lan et al., 2021). To be specific, the reaction mixture, 20 μL of 5 mg/ml CR extract solution (diluted in methanol) and 5 U/mL thrombin solution (diluted in PBS), was properly shaken for 30 s and incubated at 37°C for 40 min. Afterward, 20 μL of S-2238 was added to each well for analysis using a microplate reader (Thermo Fisher Scientific) under the mode of dynamic method. The testing was conducted for 10 min at a 6 s interval, with a detection wavelength of 405 nm. After the decomposition kinetics of the chromogenic substrates was characterized, an appropriate linear time range was selected to calculate the thrombin inhibition rate. The results showed that the linearity of 0–6 min was good, which was related to the amount of chromogen substrate added. Therefore, the inhibitory activity was determined by the slope of the linear regression between 0 and 6 min, and the inhibition rate was calculated by comparing it with the blank control.

### ELISA Evaluation

Although network pharmacology (Tao et al., 2013) and NIR modeling (Lan et al., 2021) studies have shown the presence of potential THRI in CR, the results of Tao’s network pharmacology experiment were not verified by specific *in vitro* or *in vivo* experiments. The modeling study on NIR was also essentially a spectroscopy-effect relationship experiment based on a single target, so the effect of CR on thrombin in an intracellular environment should be further evaluated. For this reason, ELISA was employed to quantify the release of thrombin.

Before the ELISA experiment, the cells in the logarithmic growth phase were digested and centrifuged and then transferred into the medium to obtain cell suspension under gentle blow. Hemacytometry was applied to count cells. The total number of the cells on the counting plate was counted under an inverted microscope, and for the cells crossing the grooves of the counting plate, only those on the left or the top of the counting chambers were counted. The calculation was as follows: the number of cells/mL= the total number of cells in five chambers ×5×10^4.^

We then collected HUVEC cells in the logarithmic growth stage and diluted them to 8×10^4^ cells/mL. The cells were then transferred to a culture plate, 100 μL/well, to gain adherent cells. After removing the medium, 100 μL of CR extract and PCR extract in 0.25, 0.5, 1.0, 5.0 and 10.0 μg/ml was added, respectively. In addition, a blank control group was established. All the samples, each with quintuple culture wells, were placed in a CO_2_ incubator for a 12 h continuous culture at 5% CO_2_ and 37°C. Then 10 μL of 5 mg/ml MTT solution was added to each well. Following another 4 h incubation at 37°C in dark, the medium was discarded, and 100 μL of DMSO solution was added to each well. The samples were then placed in a 37°C incubator for 20 min to fully dissolve the blue crystals. The absorbance value of each well was measured at 490 nm with a microplate reader. Tests were performed in triplicate, with the average taken as the result. The concentration at which the cell survival rate was above 80% was identified as the non-toxic concentration of the extract.

The HUVECs were then cultured in an endothelial cell growth medium, with fresh medium supplied every 48–72 h, and then plated in 24-well culture plates at a density of 1×10^6^ cells per well for a 24 h culture in a 5% CO_2_ incubator at 37°C. After the medium was changed, the HUVECs were respectively treated with RCR and PCR solutions for 12 h. The cell suspension was diluted with PBS (pH 7.2–7.4) to about 1 million cells/mL, and the cells were dissociated by repeated freeze-thaw procedures to release the contents, followed by 20 min centrifugation at 3,000 *g* to collect the supernatant. The absorbance of each well was detected strictly in accordance with the instructions of the FⅡα ELISA kit at 450 nm wavelength, with the blank wells used for alignment. The tests were carried out in triplicate to take the average for analysis. The data were analyzed by GraphPad Prism Version 8.4.3 (La Jolla, CA, United States). Multiple group comparison was conducted by one-way ANOVA, and *p* < 0.05 was considered statistically significant.

### Optimization of AUF Experimental Conditions

To obtain stable ultrafiltration results and maintain steady enzyme activity, the pH was set at 6.5 in this experiment, as recommended by the manufacturer.The incubation temperature was fixed at 37°C, approximately equal to human body temperature, considering the meaningful active substances should function under an *in vivo* environment. At the same time, current studies suggest that 37°C was the optimal temperature (Qin et al., 2019; Xie et al., 2021). The enzyme concentration was set at 5 U/mL due to the analytical requirements of S-2238. All experiments were carried out simultaneously to avoid the impact of repeated freeze-thawing. Based on previous research (Lan et al., 2021), the CR extract was tested, respectively, in three concentrations (1, 2.5, and 5 mg/ml; CR extract was dissolved in DMSO to 2.5, 5, and 10 mg/ml, and then diluted with culture medium) with various incubation times (30, 40 and 50 min), aiming to optimize the screening conditions. In the experimental group, CR extract was mixed with active THR, in contrast to the control group where an equal volume of methanol was used to form a mixture with THR. The absorbance value was determined at 405 nm. Besides, PBS with a volume equal to CR extract was added in each experimental group to counteract the influence of the color caused by CR extraction. We also observed whether the extract has a decomposition effect on S-2238 through the kinetic curve. All the tests were repeated three times.

### Procedures of AUF and Effective Peaks Characterization

100 μL of 2.5 mg/ml CR solution (CR extract was dissolved in methanol to prepare 25 mg/ml solution, which was diluted with PBS to 2.5 mg/ml, followed by centrifugation to obtain the working liquid) was transferred to an Eppendorf tube, then 100 μL of 5 U/mL active and inactivated THR were added for incubation at 37°C for 50 min without light. After being transferred to an ultrafiltration centrifuge tube, the samples were centrifugated at 12000 g for 15 min. The filtrate was preserved at −80°C for freezing and then transferred to a lyophilized machine to prepare the lyophilized powder. Finally, the lyophilized product was dissolved with 100 μL of LC-MS grade methanol, centrifuged to remove most of the buffer salts in the system, and then filtered by 0.22 μm microporous membrane to obtain the sample solution for LC-MS analysis.

In this experiment, compounds with an enzyme-binding rate of more than 1/3 were considered meaningful, which was calculated by the ratio of the peak area of inactive enzyme components to that of active enzyme components (Qin et al., 2015; Cai et al., 2020). It is worth noting that the use of an inactive enzyme group also played an important role in eliminating the nonspecific binding interference.

## Methods Validation

Argatroban is a known inhibitor of THR (Lewis et al., 2001). To verify whether the established method can be used to identify THRI in CR, argatroban at a variety of concentrations (20, 50, 100, 200, 500, 1,000, 2000 nM) was used to test THR activity (Wu et al., 2020). The IC_50_ of THR was determined by adding different concentrations of argatroban to 2.5 mg/mL S-2238 solution under the conditions of the optimized method. Then, the determined IC_50_ was used to further validate the efficacy of the established AUF-UPLC-MS method in which a mixture of argatroban and negative control adenosine (Zhang Q. et al., 2017) was employed as the working solution. The inhibition rate was calculated by the following formula:

$$\mathrm{Inhibition}\;\mathrm{ratio}\;\left(\%\right)=\left[{\left(\mathrm{dA}/\mathrm{dt}\right)}_{\mathrm{blank}}-{\left(\mathrm{dA}/\mathrm{dt}\right)}_{\mathrm{sample}}\right]/{\left(\mathrm{dA}/\mathrm{dt}\right)}_{\mathrm{blank}}\times 100\%$$ (1)

Where (dA/dt)_blank_ is the reaction rate of the blank group, and (dA/dt)_sample_ is the reaction rate of the sample group. The period of dA/dt is 0–6 min. The IC_50_ values of the active compounds were calculated three times in parallel at 7 concentrations. Statistical analysis and IC_50_ value calculation were conducted using GraphPad Prism Version 8.4.3 (GraphPad Software Inc., La Jolla, CA, United States).

### HPLC and LC-MS Conditions

Verification of the AUF method was performed by HPLC (Shimadzu Corporation, Japan) equipped with a vacuum degasser, binary pump, automatic sampler, and diode array detector (DAD), with combined use of an Ultimatetm XB-C18 (250 × 4.6 mm, 5 μm). According to the improved method reported in the literature (Zhang Q. et al., 2017), the mobile phase for separation was solvent A—water-acetic acid (1,000:1, v/v) and solvent B—methanol, with an elution program of 0–18 min, 15–80% B; 18–20 min, 80–15% B; and 20–25 min, 15% B. The other key conditions were set as follows: flow rate, 0.6 ml/min; DAD detection wavelength, 254 nm, and 330 nm; column temperature, 35°C; and injection volume, 10 μL.

UPLC-Q-Exactive Orbitrap/MS was performed using Ultimate 3,000 Ultra-performance liquid chromatograph and Thermo Scientific Orbitrap Fusion Tribrid Mass Spectrometer (Thermo Fisher Scientific, United States) to characterize the compounds in AUF. Waters ACQUITY UPLC BEH RP18 (2.1 mm × 100 mm, 1.7 μm) was employed for chromatographic analysis, and the mobile phases consisted of solvent A—water-acetic acid (1,000:1, v/v) and solvent B—acetonitrile. The separation followed the gradient elution program: 0–5 min: 95–75% solvent A; 5–13 min: 75–70% solvent A; 13–20 min: 70–65% solvent A; 20–35 min: 65–20% solvent A; 35–43 min: 20–10% solvent A; 43–45 min: 10–95% solvent A; and 45–48 min: 95–95% solvent A. The flow rate was 0.2 ml·min-1, with a column temperature of 25°C and an injection volume of 2 μL. The detection wavelength was set at 210, 254, and 415 nm, respectively. The MS parameters were as follows: Scanning mode was Full MS/dd MS2, and the switching mode was used in the system; Nebulizer voltage was 3.5 kV; Sheath gas pressure and aux gas pressure were 35 arb and 15 arb, respectively; Ion transport temperature and evaporation temperature were 320°C, CES was 10 eV, and the average EPI scanning spectrum was obtained when CE was 15, 35, and 45. Moreover, Orbitrap Fusion Tune and Xcalibur 4.0 were employed for mass spectrometry and data acquisition. Electrospray (ESI) ion sources were used for compounds analysis, with MS frontier 8.0 and Compound Discovery 3.1 applied to analyze the structure of the compounds.

### *In vitro* thrombin Inhibition Assays

To evaluate the thrombin inhibition ability of the compounds and verify the results of AUF-LC-MS, an *in vitro* thrombin inhibition assay was developed based on the previous studies (Lan et al., 2021). The determination was carried out on a 96-well microplate. 100 μL of 2 U/mL THR was firstly placed in micro-well at 37°C for 10 min for activating, and then the identified THR ligands were added into the pores in equal volume (diluted into 7 concentrations with buffer solution) and incubated for 50 min. After completion of incubation, 35 μL of 2.5 mg/mL S-2238 was added to each well by volley, followed by 20 min of dynamical measurement at a 6 s interval under 405 nm. The blank group and the control group followed the same procedure, except for the active enzyme or samples replaced by the denaturing enzyme and buffer in the same volume. In addition, argatroban was applied as the positive control. All assays were done in triplicate, with the average value of the inhibition ratios taken as the final results. The concentration-inhibition response curve was plotted by GraphPad Prism Version 8.4.3, and the sample concentration producing 50% inhibition (IC_50_) was calculated.

### Antithrombotic Assay in Zebrafish

To further verify whether the monomer compounds that were active *in vitro* have an equivalent *in vivo* effect on preventing or treating thrombus, we used arachidonic acid (AA) to construct zebrafish thrombus models in the antithrombotic test. Zebrafish (3pdf) selected under stereomicroscopy were randomly placed in a 24-well plate containing 2 ml of embryo culture water, 12 fish in each well. In the following procedures, they were divided into four groups to be treated with different reagents—blank group, 0.1% DMSO; model group, AA (80 μM); positive control group, a mixture of aspirin (ASP, 20 μg/ml) and AA (80 μM); and administration group, a mixture of drugs (the concentration gradient was determined by the maximum tolerable concentration) and AA (80 μM). The 24-well plate was then placed at 28°C for 1.5 h, and O-dianisidine dyeing solution was added for a 10 min staining procedure without light. 10 zebrafish from each group were randomly selected and observed under a fluorescence microscope to evaluate the staining intensity of the tail thrombosis (Sun et al., 2021). Image Pro Plus 6.0 software was used to process the images for calculation.

### Molecular Docking Analysis

One purpose of molecular docking is to explore the binding force and binding energy of molecule-enzyme conjugates, and identify the most likely target molecules from a large number of compounds for further verification, while another is to locate the sites for molecule–enzyme binding. In our experiment, the crystallographic structures of THR and argatroban were obtained from the Protein Database (PDB code 1DWC). SYBYL-X 2.0 scoring function was applied to compare the selected compounds with positive and negative drugs verified in this paper. Software was used to analyze the main and side chains of the protein and repair the parts that need to be repaired, and crystal water was removed. The protein coming with ligand argatroban was used for verification, and the optimal total score was taken as the score of the compound. To verify the binding sites of the compounds, the docking box was determined by the ligand that comes with the protein, within 5 Å. Chem 3D software was employed to construct the molecules, with the compound configuration optimized by MM2 molecular mechanics. After the ligand was hydrogenated and charged, the root of the ligand was detected to search and define the rotatable bonds. All the hydrogen atoms were added to the acceptor, followed by Gasteiger charge computation with the mergence ofnon-polar hydrogens. Besides, molecular docking coordinates were determined to increase the calculation accuracy, the maximum iteration was set as 2000 to find the lowest binding free energy. Default values were used for all parameters unless otherwise stated. Finally, semi-flexible docking was employed, and the conformation with the best affinity was selected as the final docking conformation. The molecular docking was performed using the Surflex-Dock method. The target molecules were screened according to the total score. The conformation with the lowest docking score was used for docking binding mode analysis, and docking sites were analyzed using PyMOL 2.5 and Discovery Studio 4.5 software to observe the interaction between enzymes and inhibitors.

## Results and Discussion

### ELISA Assay and AUF Condition

Although our preliminary experiments have confirmed the thrombin-inhibitory effect of CR, further study is necessary to identify the active components that play key roles in thrombin inhibition. Therefore, we conducted an ELISA assay using HUVECs endothelial cells. To optimize the AUF experimental conditions, RCR and PCR working solution at three concentrations—2.5, 5, and 10 μg/ml, were used as instructed in the maximum tolerated dose experiment. The results are shown in Supplementary Figure S1A. Compared with the blank group, the CR products showed a significant thrombin-inhibitory effect at all three concentrations, making the establishment of the AUF-LC-MS method valuable for further analysis.

In the previous experiments based on a large number of samples, it was concluded that RCR was generally stronger than PCR in inhibiting THR. Taking this into account, the RCR solution was used in this AUF experiment for condition screening. Meanwhile, the decomposition rate of S-2238 under different experimental conditions was used to characterize the inhibition rate of the enzyme. During the whole screening process, the concentration of the enzyme was fixed, so the concentration of the working solution was considered to be a key factor for the optimization of experimental conditions. As shown in Supplementary Figure S1B, under a certain incubation time, when the concentration of the working solution increased from 2.5 to 5 μg/ml, its inhibition rate elevated slightly and unsubstantially, and the increase in inhibition rate was even smaller when the concentration was 10 μg/ml, which indicated that even the active substance in the 2.5 μg/ml working solution could occupy most of the binding sites. Moreover, keeping increasing the concentration of the working solution may lead to false positive results due to the competitive binding of active substances in the system. On the other hand, incubation time also affects the binding rate of ligand and target protein. Too short incubation time is not enough for the ligand to fully interact with the target protein, thus causing difficulties in subsequent analysis. On the contrary, an overlong incubation time could compromise the experimental efficiency. Therefore, it is also necessary to choose an appropriate reaction time. In this experiment, with other conditions unchanged, significant changes, not only in enzyme activity but also in the binding ability to intrinsic ligands, were observed under different incubation times, with a result showing that the strongest enzyme activity and enzyme inhibitor-binding capability were detected at an incubation time of 50 min. By summarizing the findings as mentioned above, the optimal screening conditions were set as follows: THR concentration, 5 U/mL; incubation time, 50 min; incubation temperature, 37°C; pH value of the incubation solution, 6.5; and concentration of the working solution, 2.5 mg/ml.

### Validation of the Established Approach

Aratroban is an excellent THR inhibitor with an IC_50_ of 0.243 μM under the established conditions, therefore it was used to validate the affinity ultrafiltration operation and served as a reference for activity validation experiments. A mixture of positive control (aratroban) and negative control (adenosine) was used to verify the specificity of the affinity ultrafiltration method. The results are shown in Supplementary Figure S2. The peak of aratroban was detected at 5.974 min under 254 nm, while the curve of adenosine peaked at 9.032 min under 330 nm. The peak value of argatroban was significantly reduced in the active THR co-incubated group compared with the inactive enzyme group and blank group (the mixture of positive and negative drugs, with the same amount of enzyme diluent PBS added). Whereas, no significant difference was shown between the denatured enzyme group and the blank group. In addition, there was no change in the level of adenosine in the three groups, indicating that the established method had good specificity for THR, and the established method in the inactive enzyme group could effectively prevent non-specific binding without generating additional false positive results. The denatured enzymes were produced by boiling active enzymes in a test tube over a water bath for 20 min.

### Screening Potential thrombin Inhibitors by AUF-LC-MS

There are some disadvantages to traditional methods for enzyme inhibitors screening, such as high labor intensity, time consuming and extensive material consumption. Therefore, an affinity ultrafiltration approach based on ligand-enzyme complex was applied here to solve these problems. Subsequently, a combination with ultra-performance liquid chromatography-mass spectrometry (UPLC-MS) was developed to further refine the screening of bioactive compounds and study ligand-receptor binding properties. Hence, by directly comparing the chromatographic peak areas of active-enzyme group and inactive-enzyme group after affinity ultrafiltration, the bioactive compounds in complex plant extracts were very likely to be identified. In addition, positive and negative control groups were used to verify the reliability of the method established in this study, and to exclude the interference from other inactive components.

**FIGURE 1:**
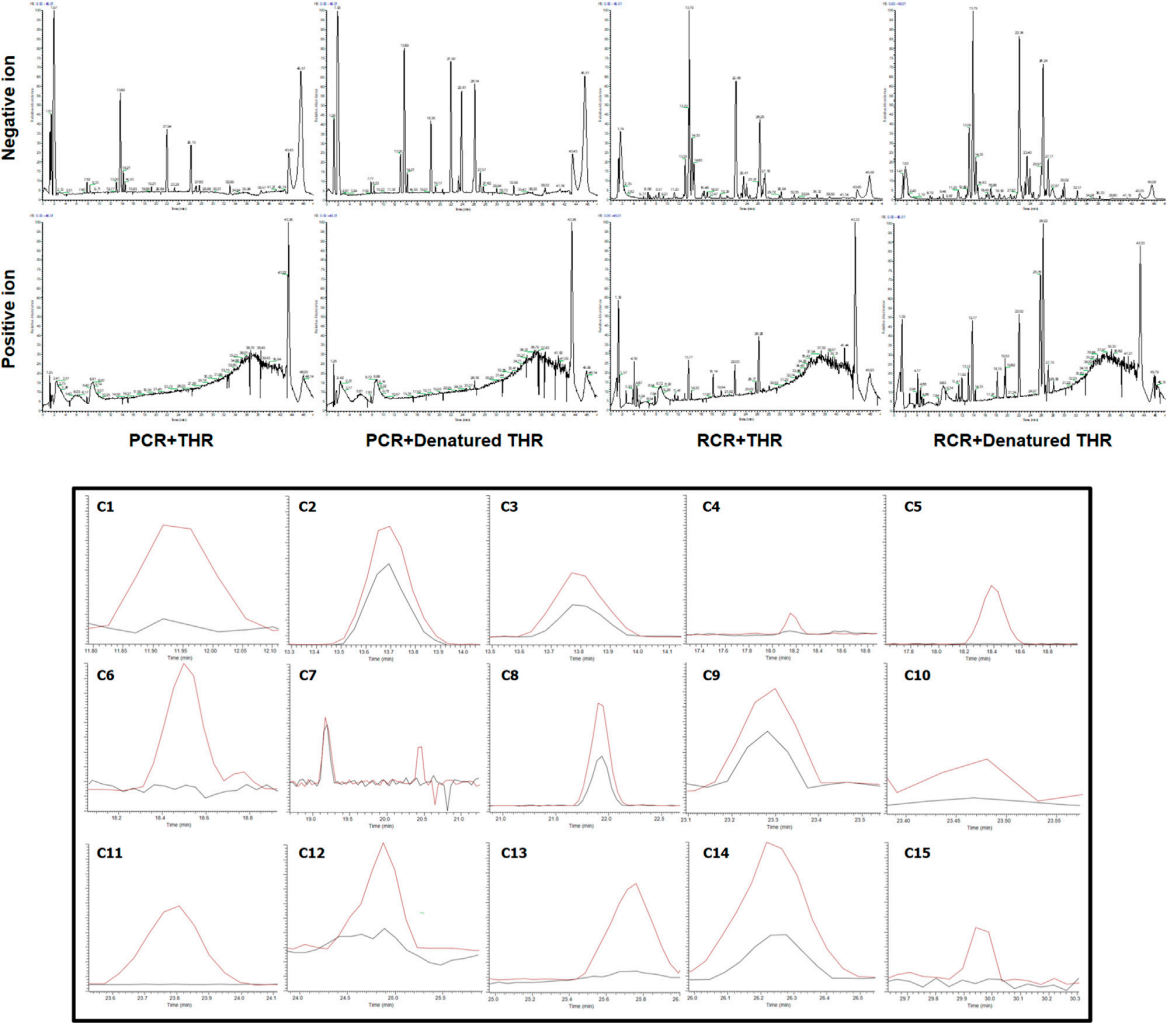
Selective ion monitoring chromatograms for screening potential THR inhibitors by combined use of affinity ultrafiltration and high-performance liquid chromatography-mass spectrometry (AUF-LC-MS). (Black line, experimental group with active enzyme; red line, control group with denatured enzyme).

**FIGURE 2:**
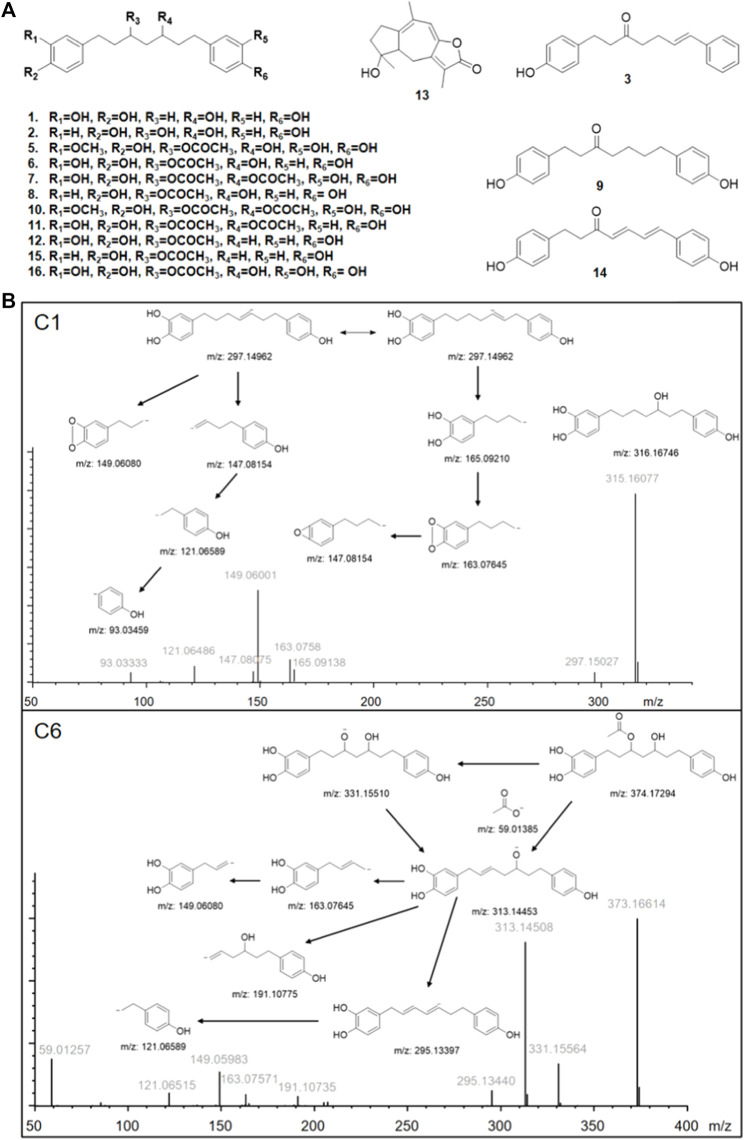
**(A)** Structures of identified bioactive compounds in *Curcumae* Rhizoma; **(B)** Proposed fragmentation pathways of C1 and C6 in negative ion mode. m/z represents the calculated theoretical mass value of the fragments; gray font represents the actual value of the measurements.

The results are shown in Figure 1. After affinity ultrafiltration screening, we found a total of 15 significantly reduced CR components with a binding rate of more than 1/3 in both the negative and positive modes. By observing the peak number of these compounds (C1—C15) based on time sequence, we found that C1, C4, C5, C6, C7, C10, C11, C12, and C13 and C15 with active enzymes disappeared after incubation, suggesting that these substances may have a strong thrombin inhibitory effect or at least a high binding ability. Yet a definite conclusion cannot be drawn because the competitive binding of the compounds in CR extract with the same targets should also be considered. Competition for the same targets will result in fewer binding sites. Under this circumstance, trace amounts of active substances are more likely to bind completely, while larger amounts of active substances will bind insufficiently due to the lack of sufficient binding sites. UPLC-Q-Exactive-MS, which has been widely used in the study of complex mixtures owing to its high resolution and mass accuracy, was employed for further identification. In this study, the structure of AUF ultrafiltrate was identified and characterized based on the real standard, accurate mass, fragment ions, and relevant literature (Zhou et al., 2016, 2018; Alberti et al., 2018; Vanucci-bacqu and Bedos-belval, 2021). The structure and other details of the compounds are shown in Figure 2A; Table 1. Based on the chemical structures, CR mainly consists of sesquiterpenes, diterpenoids, and diarylheptanoids. Amongst the 15 compounds mentioned above, 13 are diarylheptanoid structures, which means that the diarylheptanoids play a major role in CR activity. This type of structure bears some common fragmentation patterns resulting in molecular breakdown, usually at positions 1 and 7 of the skeleton and the benzene ring, or at the double bonds formed on the left and right of positions 3 and 5 due to the hydroxyl group. Moreover, acetyloxy existing on position 3 and 5 carbon atoms are also subject to the cracking law common to diarylheptanoids, which is a special structure of some compounds in this class and has characteristic fragments. In this study, compounds 1 and 6 were taken as examples for specific analytical rules, as shown in Figure 2B.

**TABLE 1:** Identification and molecular docking of active compounds in AUF step.

No.	t_R_/min	Molecular formula	m/z	Measured ions	Mass error/ppm	Fragment ions	Tentative identification	Docking scores
1	11.942	C_19_H_24_O_4_	317.1593	[C_19_H_25_O_4_]^+^	0.870	283.1332,255.1382,237.1276,227.1432,171.0805,147.0441,133.0650,107.0496	4-[5-Hydroxy-7-(4-hydroxyphenyl)heptyl]-1,2-benzenediol	7.5702
2	13.695	C_19_H_24_O_4_	315.1608	[C_19_H_23_O_4_]^-^	5.439	163.0758,149.0600,145.0807121.0649,93.0334	1,7-Bis(4-hydroxyphenyl)-3,5-heptanediol	7.0134
3	13.801	C_19_H_20_O_2_	281.1539	[C_19_H_21_O_2_]^+^	0.617	187.1120,161.0963,147.0801,133.0651,107.0497	(6E)-1-(4-Hydroxyphenyl)-7-phenyl-6-hepten-3-one	6.0415
4	18.151	-	274.2743	[-]^+^	-	247.1331,229.1226,205.0603, 183.0783,154.9904,143.0397, 102.0344	NI, Sesquiterpenes	-
5	18.412	C_22_H_28_O_7_	403.1769	[C_22_H_27_O_7_]^-^	4.490	361.1662,343.1555,328.1324,221.1183,207.1025,165.0549,163.0758,161.0601,135.0443,59.0125	4-[5-(Acetyloxy)-3-hydroxy-7-(4-hydroxy-3-methoxyphenyl) heptyl]-1,2-benzenediol	9.8642
6	18.482	C_21_H_26_O_6_	373.1662	[C_21_H_25_O_6_]^-^	4.221	331.1556,131.1451,195.1344,191.1074,136.0757,149.0598,131.0283,59.0125	4-[3-(Acetyloxy)-5-hydroxy-7-(4-hydroxyphenyl)heptyl]-1,2-benzenediol	6.7434
7	20.465	C_23_H_28_O_8_	431.1716	[C_23_H_27_O_8_]^-^	4.165	371.1505,311.1293,293.1190,249.1134,189.0916,163.0757,147.0442,59.0125	4,4'-[3,5-Bis(acetyloxy)​-1,7-heptanediyl]bis-1,2-benzenediol	10.0157
8	22.025	C_21_H_26_O_5_	357.1714	[C_21_H_25_O_5_]^-^	4.507	315.1606,297.1500,191.1073,149.0600,147.0806,145.0650,59.0125	3-Acetate-1,7-bis(4-​hydroxyphenyl)-3,5-heptanediol	9.6972
9	23.391	C_19_H_22_O_3_	297.1500	[C_19_H_21_O_3_]^-^	4.944	191.1074,149.0600,93.0334	1,7-Bis(4-hydroxyphenyl)heptan-3-one	6.2109
10	23.482	C_24_H_30_O_8_	445.1876	[C_24_H_29_O_8_]^-^	4.258	385.1662,343.1556,325.1450,189.0917,161.0601,147.0443,121.0285,59.0125	4-[3,5-Bis(acetyloxy)-7-(4-hydroxy-3-methoxyphenyl)heptyl]-1,2-benzenediol	8.5073
11	23.811	C_23_H_28_O_7_	415.1769	[C_23_H_27_O_7_]^-^	4.361	355.1555,313.1450,295.1343,189.0916,147.0443,121.0284,59.0125	4-[3,5-Bis(acetyloxy)-7-(4-hydroxyphenyl)heptyl]-1,2-benzenediol	9.2422
12	24.714	C_21_H_26_O_5_	357.1713	[C_21_H_25_O_5_]^-^	4.703	315.1606,297.1499,279.1391,163.0757,147.0442,121.0284,59.0125	4-[3-(Acetyloxy)-7-(4-hydroxyphenyl)heptyl]-1,2-benzenediol	7.0544
13	25.761	C_15_H_18_O_3_	247.1331	[C_15_H_19_O_3_]^+^	1.048	229.1225,201.1275,139.0391,123.0444,107.0860,81.0706	Zedoalactone F	4.1909
14	26.137	C_19_H_18_O_3_	293.1188	[C_19_H_17_O_3_]^-^	5.387	187.0759,119.0492,93.0334	1,7-Bis(4-hydroxyphenyl)-4,6-heptadien-3-one	6.2413
15	30.020	C_21_H_26_O_4_	341.1762	[C_21_H_25_O_4_]^-^	4.145	299.1656,281.1550,175.1122,59.0125	1,7-bis(4-hydroxyphenyl)heptan-3-yl acetate	8.0456

Docking scores are the total scores calculated by SYBYL-X 2.0. The total score of the positive drug argatroban and negative drug adenosine is 7.9147 and 5.4534, respectively. “-” represents no data, “NI” represents not identified.

The back search, which focused on the experimental activity studies of monomer molecules, was applied for these compounds in the database ZINC 15, SciFinder, and PubChem. We searched these databases for bioactive-related researches using precise molecular formulas. No bioactivity was reported for ZINC 15. Results in SciFinder and PubChem showed a great potential of diarylheptanoids in antioxidant and anti-inflammatory properties. In particular, C1 (He et al., 2011), C3, C8, C9, C11 (Li et al., 2011), C12 (Li et al., 2010) showed the inhibitory effects on nitric oxide production in lipopolysaccaride-activated macrophages. Antioxidant test results suggested that C2 (Liu et al., 2018) and C7 (Li et al., 2012) were active. C2 and C14 could increase glucose consumption in differentiated L6 myotubes (Zhang Y. et al., 2017) and exhibited good anti-tumor activity against tested tumor cell lines (HepG-2, SMMC-7721, Hela and A549) (Zhang et al., 2015). C3 had estrogen-like effects (Suksamrarn et al., 2008). C6 were evaluated for inhibitory effects on the proliferation of HH cells and HaCaT cells (Chen et al., 2015), and inhibited the release of β-hexanosidase from rat basophilic leukemic leukemia (RBL-2H3) cells to produce antiallergic activity (Matsumoto et al., 2015). C9 was reported to have strong HT-22 cytotoxicity (Jirásek et al., 2014). C14 suppressed adipocyte differentiation by inhibiting PPARγ. C/EBPα inhibits the differentiation of 3T3-L1 adipocytes (Yang et al., 2014a) and reduced pancreatic lipase activity at low concentrations (Yang et al., 2014b). In addition, C14 was also considered a selective inhibitor of COX2 (Yang et al., 2009) and has been shown to inhibit B16 melanoma cells (Matsumoto et al., 2013). C10 has been described only as a plant metabolic component and its specific activity has not been studied because it is not readily available (He et al., 2018; Wang et al., 2021). From the point of view of structure, diarylheptanoids belong to polyphenol, which means they have potentially good antioxidant activity. These identical structures were similar to curcumin to a significant extent, meaning that most studies have focused on their anti-inflammatory activity. It is worth noting that the role of these compounds in the coagulation system had not yet been reported.

### *In vitro* THR Inhibition Assays

**FIGURE 3:**
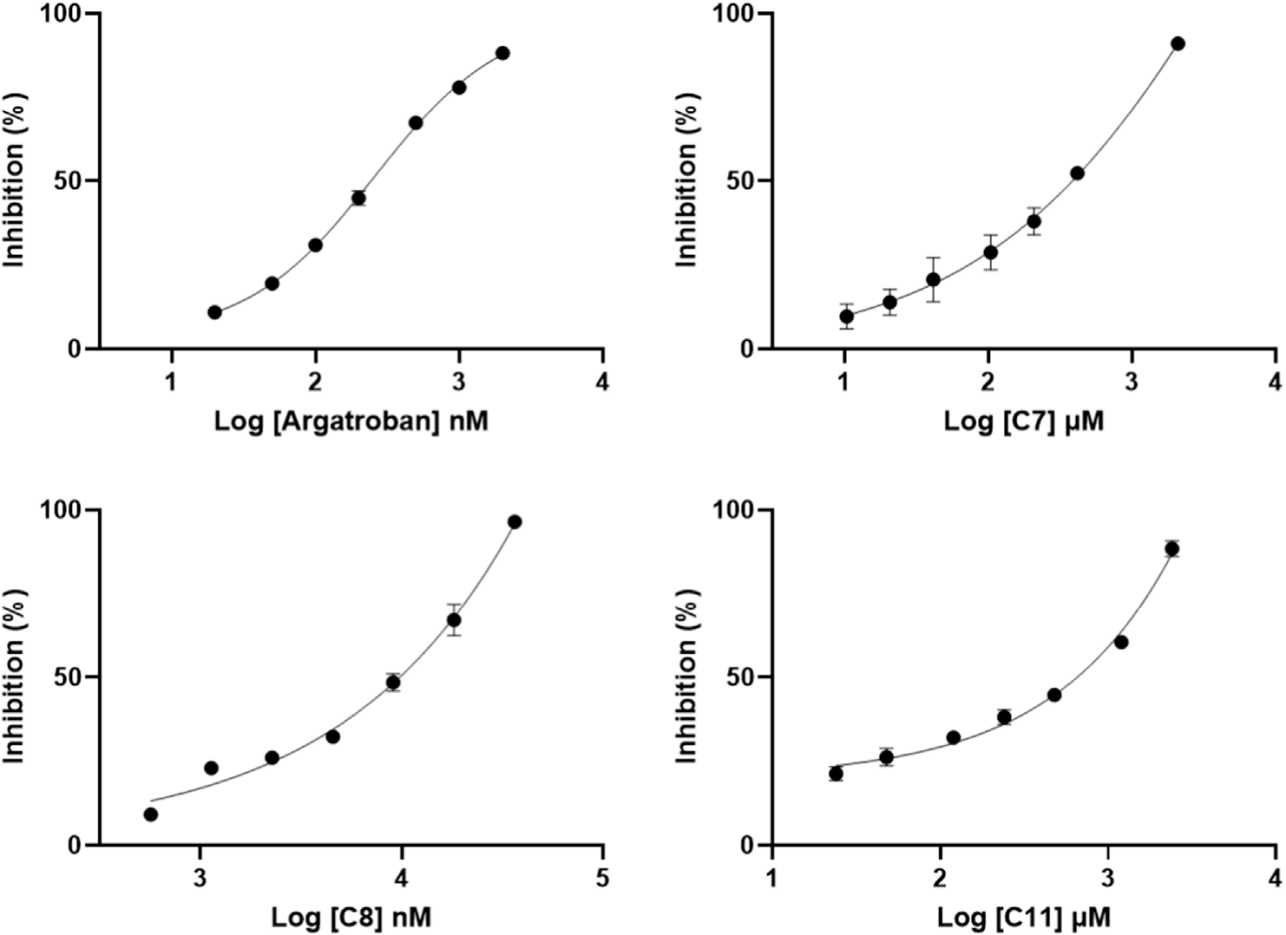
Dose-response curves of *in vitro* inhibition assays.

In order to evaluate the THR inhibitory activity of the identified compounds from CR, the enzyme activity was determined *in vitro* using compounds 7, 8, and 11 (C7, C8, and C11), as shown in Figure 3. The IC_50_ was determined by the validated method. The results showed that the IC_50_ values of C7, C8, and C11 were 358.44, 9.92, and 654.29 μM, respectively. Although the inhibition ability of these compounds is weaker than the positive drug argatroban, as extracts from natural plant species, they probably could be used as an effective and safe substitute for argatroban. The skeleton structure is very different from that of argatroban, so they are worthy of further exploration to optimize potential thrombin inhibitors. In addition, these compounds have been reported to carry a variety of other beneficial biological activities. For instance, C11 has an anti-allergic activity and can inhibit melanoma formation (Matsumoto et al., 2015). C7 and C8, as well as some other diarylheptanoid compounds, have a good antioxidant and anti-inflammatory activity (Alonso-Amelot, 2016; Jivishov et al., 2020; Nair and Paliwal, 2021; Vanucci-bacqu and Bedos-belval, 2021). Free radicals, inflammation, and thrombosis are interrelated in complex ways to each other. Inflammation can promote thrombosis in a variety of ways, and thrombogenic factors can also participate in the regulation of inflammatory response. Oxidative stress usually leads to oxidative damage of biomolecules, causing the generation of damage-associated molecular patterns (DAMPs) and the release of cytokines in the body. These changes will consequently result in increased release of cytokines and chemokines, recruitment and activation of more inflammatory cells, as well as systemic chronic inflammatory response in the body. These chronic injuries will in turn further activate the clotting pathways abnormally, and finally trigger the formation of clots (Wang et al., 2017; Yang et al., 2017; Li et al., 2018). These natural compounds with multiple synergistic biological activities show great potential to be developed as a functional dietary supplement. In brief, our experiment has demonstrated that certain diarylheptanoid compounds have a good THR inhibitory activity while at the same time having some other benefits. It is also worth mentioning that the target substance can be obtained more conveniently by affinity ultrafiltration as compared to the complex procedures of traditional methods, which require continuous purification by HPLC and activity verification of each fragment (Nongonierma and Fitzgerald, 2018). The results of the study have shown that AUF-LC-MS is a rapid and effective approach for the isolation and identification of bioactive constituents.

### Antithrombotic Activity in Zebrafish Larvae

**FIGURE 4:**
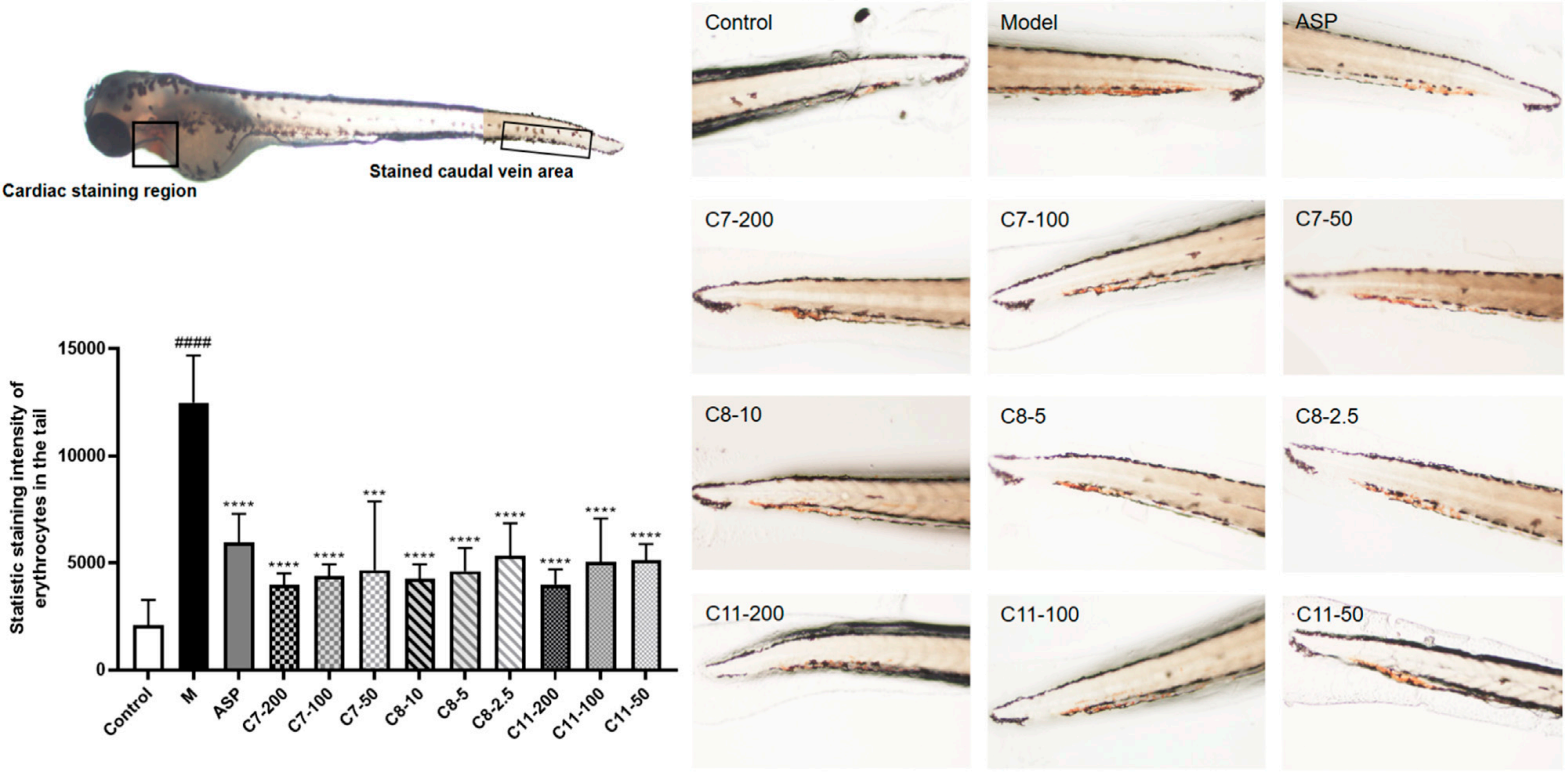
Antithrombotic activity of C7, 8, and 11 in zebrafish (*n* = 10, mean ± standard deviation). *In vivo* visualization of the erythrocytes of zebrafish tails, and statistic staining intensity of the erythrocytes in zebrafish tails of all the groups. ***p < 0.001 compared to the model (M) group. ****p < 0.0001 compared to the M group.

By using zebrafish thrombosis, we also analyzed the *in vivo* effects of the three compounds (C7, C8, and C11) that have been validated to be effective *in vitro* thrombin-inhibitory substances. The toxicity of these compounds at different concentrations was assessed within 24 h before testing using juvenile zebrafish (3 dpf), and a 100% survival rate was obtained when the concentration of C7, C8, and C11 was 200, 10, and 200 μg/ml, respectively. Three concentration gradients were employed for the administration group according to the maximum tolerated concentration, as shown in Figure 4. The staining intensity (SI) of the erythrocytes in zebrafish tails in the model group was significantly increased, implicating the successful formation of tail vein thrombosis. As a result, the administration group at the experimental concentrations showed similar antithrombotic activity as the positive group, indicating that all three compounds have *in vivo* activity to downregulate the risk of peripheral circulation blockage in AA thrombotic zebrafish. Compared with the other compounds, C8 showed a stronger effect, which was consistent with the results from *in vitro* experiments. Nevertheless, the three identified compounds all showed a significant antithrombotic activity within their safe concentration ranges (C7 less than 200 μg/ml, C8 less than 10 μg/ml, and C9 less than 200 μg/ml).

### Interaction Determination

**FIGURE 5:**
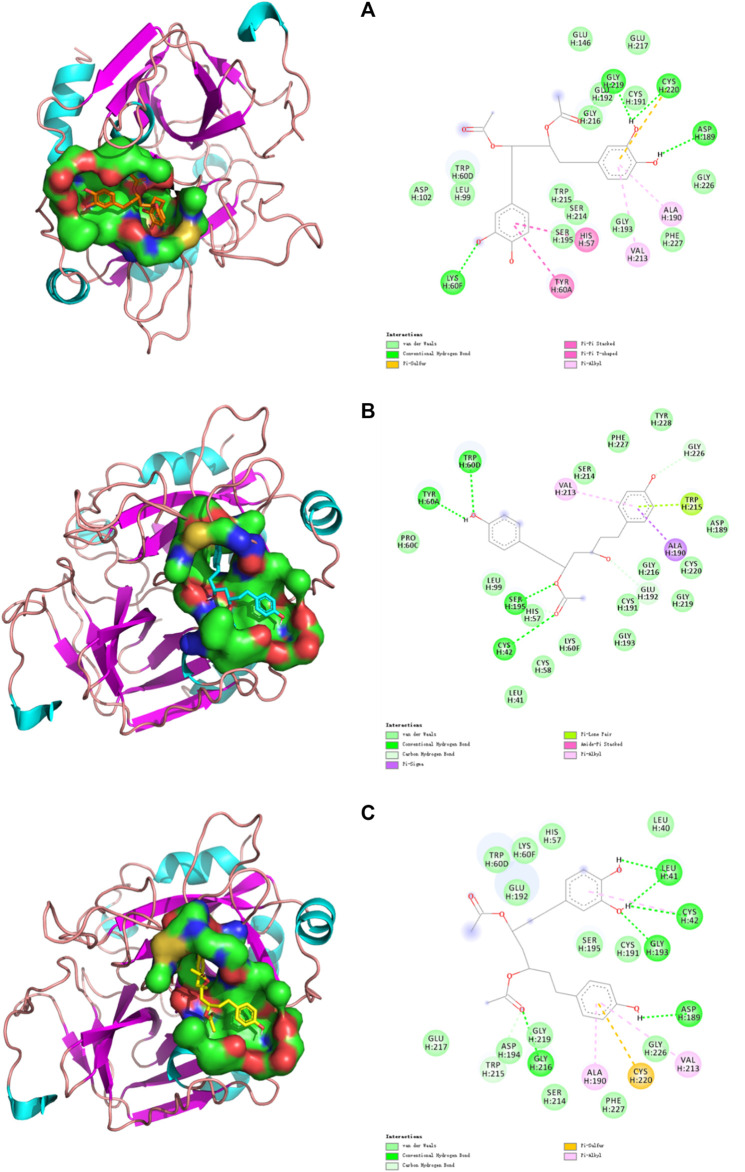
Docking models of **(A)** C7, **(B)** C8, and **(C)** C11 in THR active site. (Van der Waals are colored in light green; Hydrogen bonds are colored in deep green; Yellow, purple, deep pink, and light pink indicate hydrophobic interactions and represent Pi-Sulful, Pi-sigma, Amide-Pi Stacked, and Pi-Alkyl, respectively).

Computer-aided interactive molecular docking can be used to predict and analyze the structure-activity relationship between receptor and ligand. The three compounds that were verified *in vitro* were selected for visualized molecular docking, and the results are shown in Figure 5. In the structure of thrombin, the most critical catalytic triads (His57, Asp102, and Ser195) are located in the middle of the active site, others are in the exosite I, exosite II, and Na+ binding sites (Zheng et al., 2021). Only the active site was tested. It can be seen that the three compounds can be inserted into the catalytic active center in THR to produce a variety of forces inhibiting the enzyme activity, thereby blocking the process of thrombosis by preventing fibrinogen (FI) from turning into fibrin monomer (FⅠα). Specifically, C7 can form a Pi-Pi interaction with His57 and interacts with Asp102 and Ser195 via a weak van der Waals force. C8 can form a strong conventional hydrogen bond with Ser195 and a carbon hydrogen bond with His57. C11 can also interact with His57 and Ser195 by van der Waals force. Ser195 is located in a position that makes it act directionally and participate in nucleophilic attacks to cleavage amide bonds of substrates. His57 is involved in proton transfer and plays the catalytic role on a generalized base. In contrast, Asp102 mainly performs an adjuvant function of correctly locating His57 and has little involvement in the formation of interactions (Böhm et al., 1999). In addition, three important residues (Ala190-Cys191-Glu192) remain at the base of the active site. Ala190 can form a Pi-Alkyl interaction with the benzene rings of C7 and C11 and a strong Pi-sigma interaction with the benzene rings of C8. Cys191 interacts with the phenol hydroxyl groups of C7 and C11, and also with the hydroxyl group at position 3 of C8 to form van der Waals force. Glu192 can form van der Waals forces with C7 and C11, and carbon hydrogen bond with the hydroxyl group at position 3 of C8. Plus, some interactions demonstrated the compound’s effect on the Na^+^ binding site of thrombin, Leu99, LLE174, and Trp215 form s-hydrophobic cysts (Brandstetter et al., 1996), which facilitate the binding of aromatic residues. C7 forms van der Waals forces with Leu99 and Trp215, C8 forms van der Waals forces and Pi-Lone pair interaction with Leu99 and Trp215, respectively, while C11 forms a carbon hydrogen bond with Trp215. The Trp60D residue and S2 region extends to the active region, forming a closed hydrophobic pocket that makes it difficult to access the larger structure of the substrate. The amino acid residue forms van der Waals force interaction with C7 and C11, and strong hydrogen bonds with C8. Glu217 is the key residue of the sodium-binding allosteric site, and both C7 and C11 interacted with it (Abdel Aziz and Desai, 2018).

Table 2 shows the complete molecular docking details and the interaction of the protein 1DWC with the carrying ligand agatroban. The docking score is calculated by G_score, PMF_score, D_score, and ChemScore. The higher the score, the stronger the binding ability of the target. Among all the groups, the docking score of positive drug agatroban is 7.9147, while that of negative drug adenosine is 5.4534. Therefore, structures with a docking score of less than 5.4534 were considered less important, whereas, those with a docking score greater than 7.9147 should be valued. In our experiment, some compounds, such as C13, had a docking score of less than 5 but were also selected, possibly because their binding site was not the catalytic active site of thrombin. The results showed that the docking score of C5, C7, C8, and C11 were all higher than that of the positive drug agatroban, but this was not the case for C7, C8, and C11 in actual *in vitro* tests. The docking score represents an ideal binding ability, but in practice, factors such as solubility and toxicity of compounds also play an important role in their efficacy. In addition, as mentioned above, the amino acid residues have different degrees of importance in realizing thrombin catalytic activity but this was not considered by the molecular docking systems.

**TABLE 2:** Binding sites of the compounds and the interaction forces between monomer and thrombin.

Compounds	Hydrogen bond amino acids	Pi-interaction amino acids	Van der Waals interaction amino acids
C7	Gly219, Cys220, Asp189, Lys60F	His57, Tyr60A, Val213, Ala190, Cys220	Asp102, Leu99, Trp60D, Trp215, Ser214, Ser195, Gly193, Phe227, Gly226, Cys191, Glu192, Gly216, Glu217, Glu146
C8	Trp60D, Tyr60A, Ser195, His57, Cys42, Glu192, Gly226	Ala190, Trp215, Val213	Pro60C, Leu99, Leu41, Cys58, Lys60F, Gly193, Cys191, Gly216, Gly219, Cys220, Asp189, Tyr228, Phe227, Ser214
C11	Asp194, Gly216, Asp189, Gly193, Cys42, Leu41	Cys42, Val213, Cys220, Ala190	His57, Lys60F, Trp60D, Glu192, Leu40, Cys191, Ser195, Gly226, Phe227, Ser214, Gly219, Trp215, Glu217
Argatroban	Asp189, Gly219, Gly226, Gly216, Ser195, Glu217	Trp60D, His57, Tyr60A, Leu99, Ile174, Trp215	Asn98, Val213, Glu192, Cys191, Cys220, Ser214, Lys60F

By comprehensive analysis of the results, it can be seen that when the acetoxyl group is present at positions 3 and 5, one end of the catechol structure with more hydroxyl groups is more likely to enter the active site for binding. However, when 4-hydroxyphenyl is present at positions 1 and 7, the side with less steric hindrance at positions 3 and 5, the side near the hydroxyl group at positions 3 or 5, is more likely to enter and exert forces on multiple amino acid residues. While C7, which shows symmetry, has different binding sites at both ends, demonstrating the advantage of the catechol structure at both ends of the long chain. Based on this, a relatively ideal structure should be 4,4'-[3-(acetyloxy)-5-hydroxy-1,7-heptanediyl]bis[1,2-benzenediol], as shown in Figure 2A C16. The compound with this structure had a trace content in the samples collected in this study, which was due to a failure in considering the absorption of this compound (more than 80%) to avoid the baseline noise fluctuation. However, this compound is confirmed to be one of the diarylheptanoid compounds of CR (Vanucci-bacqu and Bedos-belval, 2021).

## Conclusion

*Curcumae Rhizoma* and its relatives are widely used as functional plants in Asia because of its rich existence in the beneficial pigment curcumin and the evident effect of promoting blood circulation and removing blood stasis. In our study, an AUF-LC-MS method based on THR affinity was successfully established for rapid, efficient, and targeted screening and identification of the bioactive compounds in CR. A total of 15 active compounds (13 diarylheptanoid, 1 diterpenoids, and 1 sesquiterpenes) were found and identified, with some of them verified to be effective in in vitro THR inhibition experiments. Further analysis was conducted by molecular docking to study the structure-activity relationship of the compounds, and the *in vivo* antithrombotic activity of these compounds was also evaluated using a zebrafish model. The results confirmed the discovery of a new biological activity--thrombin inhibition--of specific diarylheptanoid structures, suggesting that this natural skeleton may have the potential to be further developed and modified as THRI. Additionally, as mentioned above, some studies have shown that the ingredients verified in this paper have antioxidant and anti-inflammatory effects. Since the process of thrombotic disease is closely related to oxidative stress and inflammatory response as known to all, treatments consider that in addition to their direct role in the clotting pathway, antioxidants, and anti-inflammatory work together to produce antithrombotic activity. For this reason, these substances have shown great potential as a dietary supplement to protect the cardiovascular system. This method can accelerate and simplify the study of bioactive compounds in natural products. The analysis of its bioactive components is also helpful for its quality evaluation and clinical application.

## Data Availability

The original contributions presented in the study are included in the article/Supplementary Material, further inquiries can be directed to the corresponding authors.
