# Supplementary material for: Identifying of Anti-Thrombin Active Components From Curcumae Rhizoma by Affinity-Ultrafiltration Coupled With UPLC-Q-Exactive Orbitrap/MS
Source: Front Pharmacol. 2021 Dec 10;12:769021. doi: 10.3389/fphar.2021.769021 (PMC8703108; doi:10.3389/fphar.2021.769021)
Supplement: Supplementary file 1 [file Table1.DOCX]

Screening of anti-thrombin active components from Curcumae Rhizoma by affinity-ultrafiltration coupled with UPLC-Q-Exactive Orbitrap/MS

Supplementary Material

**Zhenwei Lan^1^**^†^**, Ying Zhang^1^**^†^**, Sun Yue^1^**^†^**, Lvhong Wang^1^, Yuting Huang^1^, Hui Cao^2^*, Shumei Wang^1^*, Jiang Meng^1^***

^1^ School of Traditional Chinese Medicine, Guangdong Pharmaceutical University; Key Laboratory of Digital Quality Evaluation of Chinese Materia Medica, State Administration of Traditional Chinese Medicine (TCM); Engineering Technology Research Center for Chinese Materia Medica Quality of Universities in Guangdong Province, Guangzhou, 510006, Guangdong, China.

^2^ College of Pharmacy，Jinan University, Research Center for Traditional Chinese Medicine of Lingnan (Southern China)，Jinan University, Guangdong Provincial Key Laboratory of Traditional Chinese Medicine Informatization, Guangzhou 510632, China

^†^These authors have contributed equally to this work and share first authorship.

*Corresponding author: Prof. Hui Cao, Email: [Kovhuicao@aliyun.com](mailto:Kovhuicao@aliyun.com); Shumei Wang, Email: [shmwang@sina.com](mailto:shmwang@sina.com); Prof. Jiang Meng, Department of Traditional Chinese Medicine, Guangdong Pharmaceutical University, Guangzhou, 510006, China, E-mail: [jiangmeng666@126.com](mailto:jiangmeng666@126.com), Fax: +86-020-2039352174.


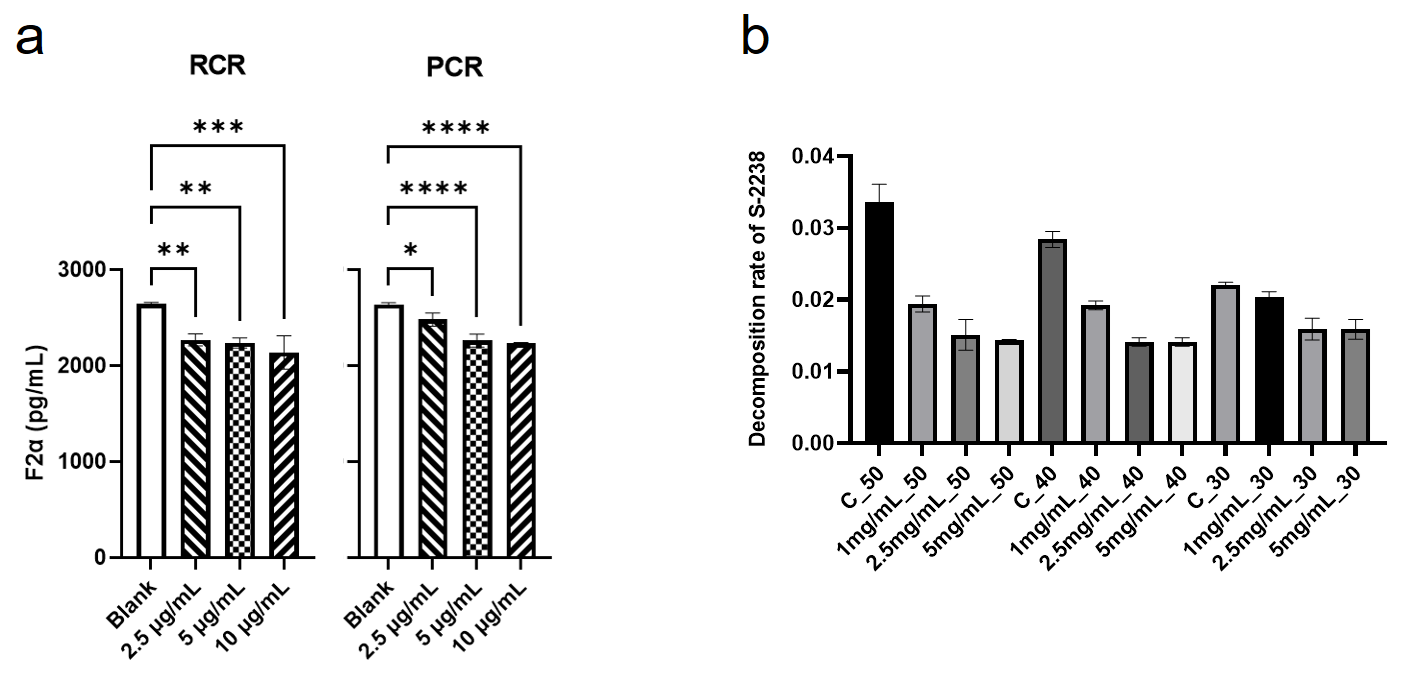


**Figure S1**. a) ELISA assay on HUVECs endothelial cells, *p < 0.05 compared to the Blank group (B). **p < 0.01 compared to the B group. ***p < 0.001 compared to the B group. ****p < 0.0001 compared to the B group; b) Effects of 30, 40 and 50 min incubation at 37 °C on groups administered with different concentration. C represents the control group, and the number after concentration represents incubation time.


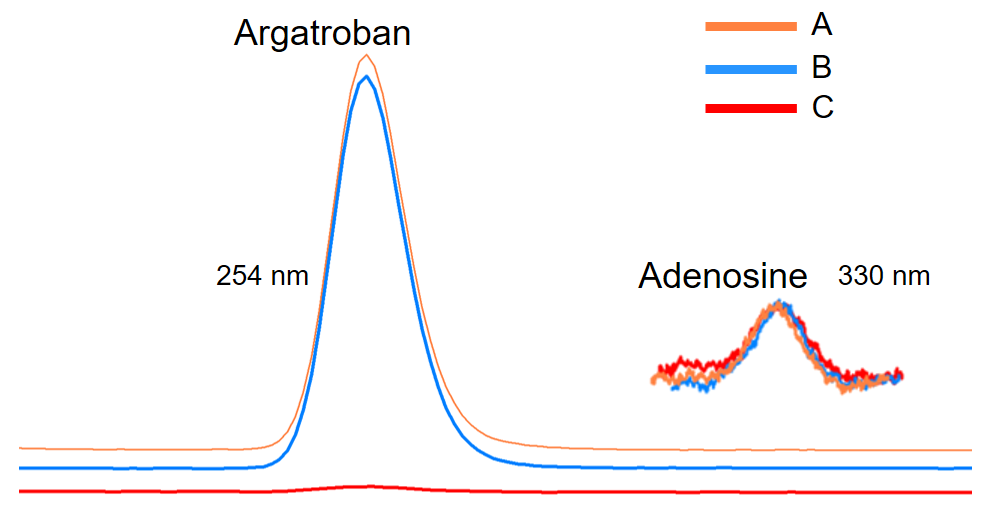


**Figure S2**. The feasibility of the established method was verified by the mixed working solution (MWS) of positive and negative drugs specifically bound to thrombin: A) the filtrate (mixture of MWS and enzyme diluent) obtained through the ultrafiltration membrane under established method; B) the filtrate (mixture of MWS and denatured enzyme) obtained through the ultrafiltration membrane under established method; C) the filtrate (mixture of MWS and active enzyme) obtained through the ultrafiltration membrane under established method.
